# mNGS facilitates the accurate diagnosis and antibiotic treatment of suspicious critical CNS infection in real practice: A retrospective study

**DOI:** 10.1515/biol-2022-0578

**Published:** 2023-03-03

**Authors:** Li Feng, Jiaxin Chen, Qiuyan Luo, Miao Su, Peisong Chen, Rong Lai, Cunzhou Shen, Hongyan Zhou, Haiyan Wang, Xunsha Sun, Ling Chen, Han Xia, Huiyu Feng

**Affiliations:** Department of Neurology, Neurological Intensive Care Unit, The First Affiliated Hospital, Sun Yat-Sen University; Guangdong Provincial Key Laboratory of Diagnosis and Treatment of Major Neurological Diseases; National Key Clinical Department and Key Discipline of Neurology, No. 58 Zhongshan 2nd Road, Guangzhou, 510080, China; Department of Laboratory Medicine, The First Affiliated Hospital, Sun Yat-sen University, Guangzhou 510080, China; Department of Scientific Affairs, Hugobiotech Co., Ltd., Beijing 100176, China

**Keywords:** mNGS, severe CNS infection, pathogen identification, empirical antibiotic treatment, accurate adjustment

## Abstract

Whether metagenomic next-generation sequencing (mNGS) could benefit patients with suspected severe central nervous system (CNS) infection in terms of diagnosis and antibiotic treatment remains unknown. We retrospectively analyzed 79 patients with suspected CNS infection and undertook mNGS. The value of mNGS was investigated in terms of identification of pathogen and guidance for the adjustment of antibiotic treatment. The relationship between the time of initiating mNGS since onset and the Glasgow Outcome Scale (GOS) score after 90-day follow-up were analyzed. Fifty out of 79 cases with suspicious severe CNS infection were finally diagnosed. Despite previous routine laboratory tests, mNGS further promoted the accurate identification of pathogens in 23 cases (47.9%). The sensitivity, specificity, and accuracy of mNGS test in this study were 84.0, 79.3, and 82.3%, respectively. Furthermore, mNGS facilitated the adjustment of empirical antibiotic treatments in 38 cases (48.1%). The time of taking mNGS since onset had an insignificant weak positive correlation with GOS after 90-day follow-up (*r* = −0.73, *P* = 0.08). mNGS facilitated the accurate identification of pathogens in suspicious severe CNS infections and promoted the accurate antibiotic therapy even empirical antibiotics were administrated. It should be taken as early as possible to improve the clinical outcome of patients with suspicious severe CNS infection.

## 1 Introduction

The central nervous system (CNS) infection is common throughout the world [1]. So far, there are more than 100 microbes which can cause encephalitis or meningitis, and the list keeps growing [2]. The clinical manifestations of CNS infection, including headache, fever, seizures, and alternation of consciousness, are nonspecific, overlapping with that of other CNS diseases, such as autoimmune encephalitis [3]. On the other hand, the sensitivity and specificity of conventional laboratory tests, which usually include cerebrospinal fluid (CSF) analysis, polymerase chain reaction (PCR), CSF culture, and brain biopsy, are unsatisfying. Various factors, such as the increasing pathogenic microbes compared with limited definitive tests, the rare pathogens that spread widely due to globalization, or just the inaccessibility to enough volume of CSF or brain tissue, bring difficulty in identifying pathogens and providing pathogen-specific antibiotic [4,5]. The pathogen of acute meningeal encephalitis is not identified in about 50% cases [5,6].

Timely and accurate diagnosis and treatment are essential to improve the outcome of patients with severe CNS infections. Metagenomic next-generation sequencing (mNGS) is a novel but promising method assisting clinicians to identify pathogens in a rapid target-independent manner. It allows simultaneous and unbiased identification of all microbes with a small volume of single sample [7]. So far, mNGS has been gaining increasing attention of researchers and success in disclosing the rare or unexpected pathogens in piles of infection [5,8,9]. A lot of studies have verified its utility in CNS infection. The overall positive detection rate is approximately 15.7–57% in CNS infection, with a sensitivity of 73–92% and a specificity of 96–99%, depending on the pathogens [1,5,6,8,10]. Although the reliability of mNGS in identifying pathogens of CNS infection is gradually recognized, and the fact that mNGS facilitates the diagnosis and treatment of rare pathogens in CNS infections has been acknowledged, whether the application of mNGS promotes treatment of CNS infection in real world is rarely discussed [5,6]. Empirical antibiotic treatment covering most common pathogens is usually administrated before mNGS in real clinical scenario; this may limit the application of mNGS if the mNGS detection contributes little to treatment adjustment. Therefore, it is necessary to investigate the strength of mNGS in promoting not only diagnosis but also treatment in real clinical scenario.

In this study, we retrospectively analyzed 79 cases admitted to our neurological intensive care unit (NICU) with suspected severe CNS infection during the past 5 years. Before mNGS test, all patients received empirical antibiotic treatment toward pathogens judged by clinical symptoms and routine laboratory testing. In this study, the mNGS significantly facilitated the identification of pathogens as well as promoted the accurate antibiotic treatment profoundly.

## 2 Methods

### 2.1 Subjects

Patients admitted to NICU in the First Affiliated Hospital of Sun Yat-Sen University from January 2017 to December 2021 due to suspicious severe CNS infection were enrolled in this study. The inclusion criteria were set as (1) at least one of the following symptoms: fever (>38℃), headache, nausea/vomiting, seizure, meningeal irritation, local neurological dysfunction, and conscious disorder and (2) at least one of the following changes: the infectious CSF and/or the infectious changes on images. The exclusion criteria included: (1) age <18 or >80 years; (2) refused lumbar puncture and/or mNGS of CSF during hospitalization; and (3) pre-existing evidence suggesting other etiology (i.e., positive antibody for autoimmune encephalitis).


**Informed consent:** Informed consent has been obtained from all individuals included in this study.
**Ethical approval:** The research related to human use has been complied with all the relevant national regulations, institutional policies and in accordance with the tenets of the Helsinki Declaration and has been approved by the ethical review committee of the First Affiliated Hospital of Sun Yat-Sen University.

### 2.2 Chart review and data collection

The initial diagnoses at registration and the final clinical diagnoses for the enrolled patients were verified by retrospective, in-depth chart review independently performed by two neurologists (Dr FL and Dr FH or Dr CL) and one infectious-disease physician and microbiologist (Dr CP). Any discrepancies in the determination of etiology were resolved by direct communication with treating physicians or by mutual consensus. For cases whose diagnoses meeting the inclusion criteria mentioned above, the general information of patients, including the age, gender, time of onset and registration, main symptoms, physical signs, imaging findings as well as the scores of Glasgow coma scale (GCS) and Acute Physiology and Chronic Health Evaluation II (APACHE Ⅱ) at registration in NICU, was collected. The medical process since onset, the time and details of CSF analysis, the results of PCR and specific antibody against pathogen, and the time and results of mNGS were also recorded. Empirical antibiotics based on initial diagnosis (virus, bacteria, tuberculosis, fungi, parasite, and unidentified pathogens according to the clinical symptoms and routine tests) before mNGS were recorded. For the adjustment of antibiotic regimen after mNGS, the “Add” referred to adding a new sort of antibiotic against another pathogen or a new relevant drug according to revised diagnosis, the “De-escalation” meant degradation or suspension of an antibiotic, while the “Modification” was defined as modifying the antibiotic but still against the same spectrum of pathogen. The overall time and cost of hospitalization, intensive care unit (ICU) duration, and the score of Glasgow Outcome Scale (GOS) after 90-day follow-up were also included.

### 2.3 Follow-up

The follow-up was carried out on the 90th day after discharge via outpatient clinic, online medical consultation service, or telephone. GOS of the patients with follow-up was recorded to evaluate their disability.

### 2.4 mNGS of CSF

About 2 mL of CSF was collected and then transmitted on dry ice for PACEseq mNGS detection (Hugobiotech, Beijing, China). The cells in CSF were removed by centrifugation and the supernatant was collected for the subsequent extraction. Cell-free DNA from CSF was extracted using QIAamp DNA Micro Kit (QIAGEN, Hiden, Germany) according to the manufacturer’s instructions. DNA libraries were constructed using QIAseq™ Ultralow Input Library Kit for Illumina (QIAGEN, Hiden, Germany) according to its manual. The quality of constructed libraries was assessed using Qubit (Thermo Fisher, Waltham, USA) and Agilent 2100 Bioanalyzer (Agilent Technologies, Palo Alto, USA). The qualified DNA libraries were sequenced on a Nextseq 550 platform (Illumina, San Diego, USA). Negative controls (sterile deionized water) and positive controls (synthesize fragments with known quantities) were set for each batch of experiments using the same wet lab procedures and bioinformatics analysis as the clinical samples. For the bioinformatics analyses, adapters, short, low-quality, and low-complexity reads were removed from the raw data of each library. The human DNA reads were also filtered out by mapping to human reference database (hg38). The remaining reads were aligned to the Microbial Genome Databases (ftp://ftp.ncbi.nlm.nih.gov/genomes/) using Burrows-Wheeler Aligner software. The reads number and reads per million (RPM) of each detected pathogen was calculated. For detected microbes, including bacteria (*Mycobacteria* excluded), fungi (*Cryptococcus* excluded), and parasites, a positive mNGS result was given when its coverage ranked top10 of the same kind of microbes and absent in the negative control (“No template” control, NTC) or when its ratio of RPM between sample and NTC (RPM_sample_/RPM_NTC_) > 10 if RPM_NTC_ ≠ 0. For viruses, *Mycobacteria*, and *Cryptococcus*, a positive mNGS result was considered when at least 1 unique read was mapped to species level and absent in NTC or RPM_sample_/RPM_NTC_ >5 when RPM_NTC_ ≠ 0.

### 2.5 Statistical analysis

SPSS 17.0 was used to analyze the relevant data. Descriptive statistics were used for demographic information as Mean ± SD. The data that were not normally distributed were described as [*M*(*Q*_*L*_, *Q*_*U*_)]. The proportion of specified group was presented as percentage. The relationship between the timing of mNGS and clinical outcome was tested by Spearman’s correlation analysis.

## 3 Results

### 3.1 General data

A total of 79 cases (male:female = 57:22) with initial diagnosis of suspicious severe CNS infection at admission were enrolled into this study during 2017–2021. The age of these patients was 43.29 ± 19.76 years old; their GCS and APACHE Ⅱ score at the registration of NICU were 6.41 ± 3.54 and 16.87 ± 4.93, respectively. The average time of starting empiric antibiotics administration was 10.80 ± 13.44 days since onset. The average time of taking the first mNGS since onset was 18.61 ± 15.64 days. The general information and clinical characteristics of the 79 patients are listed in Table 1.

**Table 1:** Demographic and clinical characteristics of the 79 patients

Characteristic	Value
Age – *M ±* SD	43.29 ± 19.76
Male:female – *n*	57:22
Syndrome – *n* (*%*)	
Meningitis	7 (8.9)
Encephalitis with or without meningitis	64 (81.0)
Myelitis with or without meningitis	8 (10.1)
Exacerbation of chronic condition – *n* (%)^†^	9 (11.4)
Immunocompromised – *n* (%)	12 (15.2)
Solid-organ transplant	2 (2.5)
Chemotherapy	6 (7.6)
Immunosuppression for non-neoplastic condition	4 (5.7)
GCS at ICU registration – *M ±* SD	6.41 ± 3.54
APACHE Ⅱ at ICU registration – *M ±* SD	16.87 ± 4.93
Mean length of stay (day) – *M*(*Q*_*L*_, *Q*_*U*_)	
In hospital	32 (21,56)
In ICU	18 (8,28)
Median no. of days after onset that antibiotics were used against CNS infection (day) – *M*(*Q*_*L*_, *Q*_*U*_)	7.5 (2.75, 14)
Median no. of days after onset that CSF was collected for metagenomic NGS (day) – *M*(*Q*_*L*_, *Q*_*U*_)	15 (8,27)
Mean total cost (U.S.$) – *M*(*Q*_*L*_, *Q*_*U*_)	29,618 (13,341, 50,720)

^†^Symptoms had been present for more than 4 weeks.

Among the 79 patients, mNGS of CSF provided 48 positive results and 31 negative results. The empirical judgment on pathogen has been revised in 46 (58.2%) cases by mNGS, among which there were 23 positive mNGS results and 23 negative results. 50 patients were finally diagnosed with severe CNS infections. According to the final diagnosis, the number of cases with true positive, false positive, true negative, and false negative results by mNGS was 42, 6, 23, and 8, respectively. The sensitivity of mNGS in identifying pathogens of severe CNS infection shown in this study was 84.0%, while the specificity was 79.3%. The accuracy of mNGS in this study was 82.3%.

**Figure 1:**
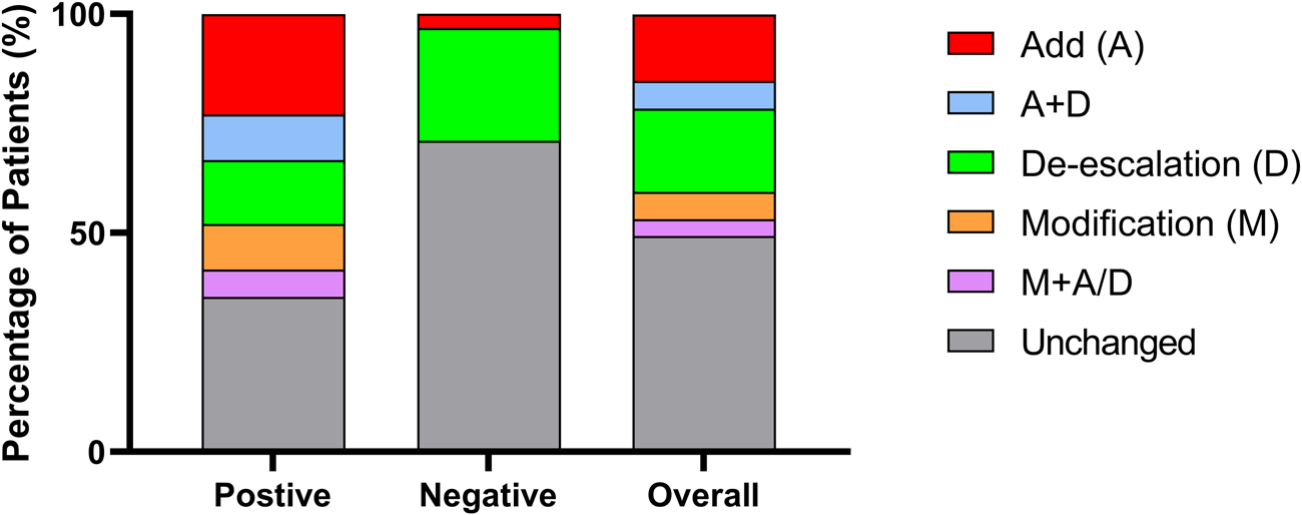
The adjustment of antibiotic treatment of severe CNS infection owing to mNGS results. The separated squares with different color of each column represented the adjustment of antibiotics according to mNGS results.

There were 38 patients (48.1%) went through adjustment of antibiotics based on the mNGS results. Antibiotics were added in 18 cases (22.8%), de-escalated in 20 cases (25.3%), and modified in 7 cases (8.7%) (Figure 1). Five patients received addition and de-escalation of antibiotic treatment simultaneously, and two patients went through modification simultaneously with addition or de-escalation.

### 3.2 Cases with positive mNGS detection

**Figure 2:**
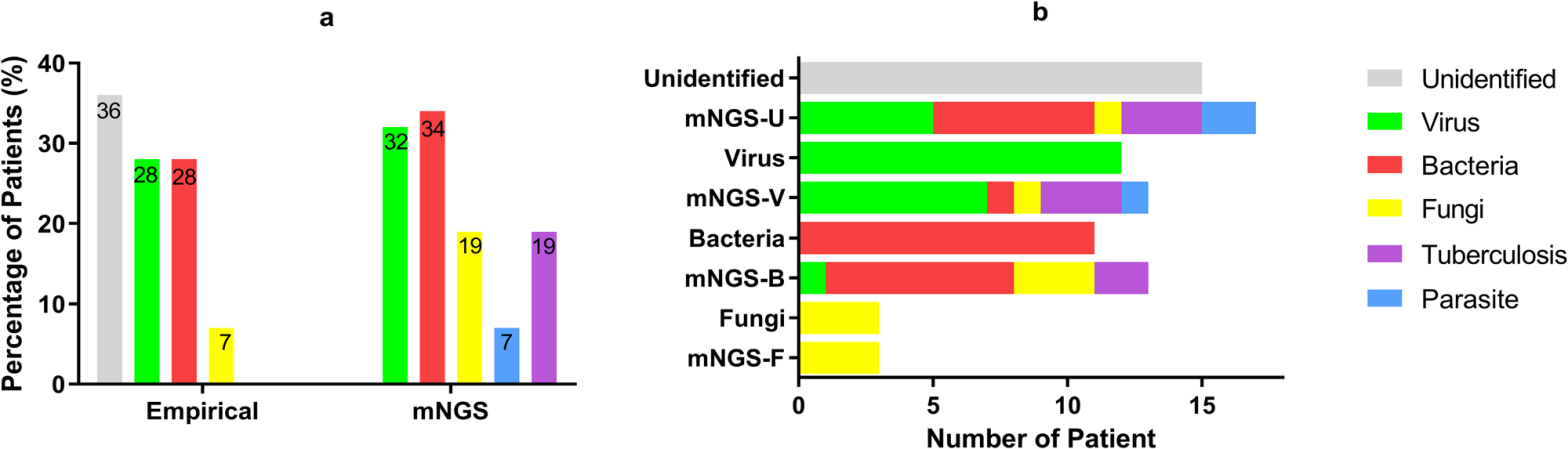
The spectrum of pathogens among patients with positive mNGS results. (a) The pathogen spectrum judged by empirical judgment and mNGS, respectively. The number represented the percentage of pathogens among the positive mNGS cases by empirical judgment and mNGS, respectively. (b) The judgment on the spectrum of pathogens has been profoundly changed by mNGS. The rows named unidentified/virus/bacteria/fungi represented the case number of each pathogen spectrum judged by clinical data and routine CSF tests before mNGS, the rows named mNGS-U/V/B/F represented the case number of each pathogen identified by mNGS.

There were 48 cases (M:F = 33:15) detected with positive mNGS results in this study, and 42 were verified as CNS infection. Compared with the empirical judgment on pathogen, mNGS has helped to determine pathogens in 15 cases with unidentified clinical and laboratory characteristics and thoroughly changed the categories of pathogens in 6 cases in further, mainly from virus or bacteria to other pathogens. It also helped to expand the pathogen spectrum in additional 2 cases. Totally, mNGS promoted the pathogen identification in 47.9% cases (23) when the result was positive. More details are in Figure 2 and Table 2.

**Table 2:** The diagnosis and treatment adjustment of patients with positive mNGS

Pathogens empirically supposed		Pathogens identified by mNGS		Treatment adjustment	
Species	No.	Species	No.	Adjustment	Percentage
Virus-U^a^	12	Virus^b^	7	Add	25
		*Strongyloides stercoralis*	1	De-escalation	66.7
		TB	3	Unchanged	25
		*Norcardia*	1		
		*Mucor*	1		
Bacteria^c^	12	Bacteria^d^	8	Add	54.6
		CMV	1	De-escalation	18.2
		TB	2	Modification	18.2
		Fungi	3	Unchanged	27.3
Fungi	3	*Cryptococcus*	2	Modification	33.3
		*Coccidioides*	1		
Unidentified^e^	15	Virus^f^	6	Add	46.7
		Bacteria^g^	6	De-escalation	13.3
		TB	3	Modification	26.7
		*Aspergillus funigatus*	1	Unchanged	26.7
		*Angiostrongylus cantonesis*	1		
		*Nargleria fowleri*	1		

^a^ *Strongyloides stercoralis* co-existed with HV-5 (1), CMV & HV-α (1); ^b^CMV, HV-α, HV-δ, HV-ε, β-HPV-1, HPV-5, HZV; ^c^ *Streptococcus pneumoniae* coexisted with Aspergillus; CMV coexisted with Listeria monocytogenes; ^d^ *Listeria monocytogenes, Norcardia*, *Streptococcus intermedius, Streptococcus pneumoniae, Staphylococcus, Roseomonas mucosa*; ^e^ *Acinetobacter baumannii* co-existed with *Stenotrophomonas maltophilia*; ^f^HV-α, EBV, CMV, Parvovirus; ^g^ *Klebsiella pneumoniae, Norcardia, Acinetobacter baumannii, Stenotrophomonas maltophilia, Streptococcus suis*, Abbreviations: U: unclassified; CMV: Cytomegalovirus; HV: Herpus virus; HPV: Human paliiomavirus; HZV: Herpes zoster virus; TB: tuberculosis; EBV: Epstein-Barr virus.

mNGS promoted the optimization of antibiotic treatment in 29 out of 48 with positive mNGS results (60.4%), including 12 (25.0%) with de-escalation of antibiotics, 7 (14.6%) with modification toward more targeted antibiotics, and 17 (35.4%) with added antibiotics. In addition, mNGS results facilitated the decision of surgery in 2 cases (4.2%). Thirteen cases (39.6%) remained their antibiotic treatment, because the initial empirical treatment had covered the pathogens identified by mNGS (Figure 1), or simultaneous infection of other organ (mostly pneumonia) was considered. Five of the six false-positive cases remained their previous antibiotic treatments due to infection of other organs. Only one went through de-escalation because of the absence of virus in mNGS result.

There were 28 CSF samples sent for culture or direct microscopy, and only to get 5 (17.8%) positive results. Among the five positive samples, three suggested cryptococcus, and one suggested fungus without specific specie. Another one was considered contamination. The three cryptococcus-positive CSF samples were the only samples with which the conventional test identified pathogens prior to mNGS and promoted the adjustment of antibiotic regimen.

Six (12.5%) cases were finally identified as mNGS false positive, with the final diagnosis of acute disseminated encephalomyelitis (ADEM) (2), anti-NMDA-receptor encephalitis (anti-NMDARE) (1), septic encephalopathy (1), autoimmune encephalitis with negative antibody (1), and cerebral bleeding (1). The false positive rate of mNGS was 7.6%. The clinical characteristics and mNGS discoveries of six cases are listed in Table 3.

**Table 3:** The information of patient with false-positive mNGS

mNGS		Clinical diagnosis		Treatment
Pathogen identified	No.	Final diagnosis	No.	
HV-4	1	ADEM	2	Unchanged
*Klebsiella pneumoniae*	1			
*Pseudomonas stutzeri*	1	Autoimmune encephalopathy with negative antibody	1	Unchanged
*Micrococcus luteus*	1	NMDARE	1	Unchanged
CMV	1	Septic/metabolic encephalopathy	2	Unchanged
HPV	1			

### 3.3 Cases with negative mNGS detection

Thirty-one patients (M:F = 24:7) were tested with negative mNGS results. The mNGS helped to revise the etiology of 74.2% (23) cases with negative findings. The 23 cases with true negative results were eventually diagnosed as AE (1), probable AE (4), possible AE with negative antibody (11), ADEM (2), cerebrovascular disease (1), fatal familiar insomnia (1), meningeal carcinoma (1), hypoxic ischemic encephalopathy (1), and metabolic encephalitis (1). Eight (25.8% of the cases with negative mNGS) cases were considered mNGS false negative, among which 2 cases were finally diagnosed with tuberculosis via the second mNGS and Xpert test, one was diagnosed with Japanese B encephalitis infection by specific antibody test and MRI, and five cases were diagnosed with abortive bacterial infection based on the disclosure of lesion in the inner ear, characteristics of CSF, and the efficacy of antibiotic therapy (Table 4).

**Table 4:** The diagnosis of patients with false negative mNGS

Patient no.	Age (years)	Gender	Syndrome	Pathogen	Approaches to diagnosis
A	39	M	Meningitis	Bacteria	Discover the inner ear abscess
B	48	M	Meningoencephalitis	Bacteria	The clinical and CSF characteristics, effective antibiotic treatment
C	56	M	Encephalitis	Bacteria and/or virus	
D	59	M	Encephalitis	Bacteria	
E	56	M	Encephalitis	Tuberculosis	The second CSF mNGS and specialist consultation
F	14	M	Encephalitis with myelitis	Bacteria	Discover the inner ear abscess
G	34	M	Meningoencephalitis	Japanese B virus	Specific antibody test
H	47	M	Encephalitis	Tuberculosis	CSF-Xpert, specialist consultation, effective anti-TB treatment
I	19	M	Meningitis	FFI	Genetic screen and PCR (D178N mutation)

FFI: fatal familiar insomnia.

Eight cases (25.8%) went through degradation or even terminating the initial empirical antibiotic treatment according to the negative mNGS results. One (3.33%) case was added with high-dose steroid intravenous impulse due to the absence of infection verified by the negative mNGS (Figure 1). There were 22 (71.0%) cases continuing their antibiotic regimen, mainly because simultaneous infection of other organs (such as pneumonia) or the worry about CNS infection with unknown virus. Besides, 5 out of the 22 cases received other antibiotics, which did not target CNS infection after a sufficient dosage and duration of initial empirical antibiotics.

### 3.4 Follow-up

**Figure 3:**
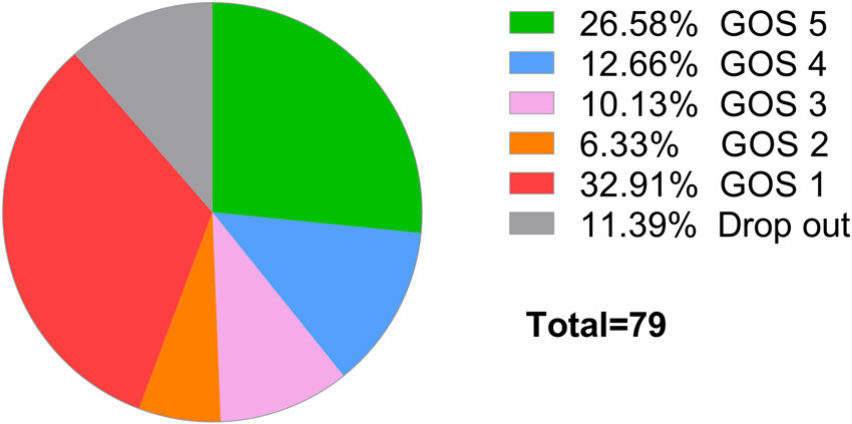
The GOS score of patients with suspected severe CNS infection at the 90th day after discharge.

Totally, 70 patients were followed up and 9 were lost (Figure 3). The total mortality was 37.1% and the total recovery ratio was 21%. The average GOS score of patients with severe CNS infection was 2.93 ± 1.71 on the 90th day after discharge.

**Figure 4:**
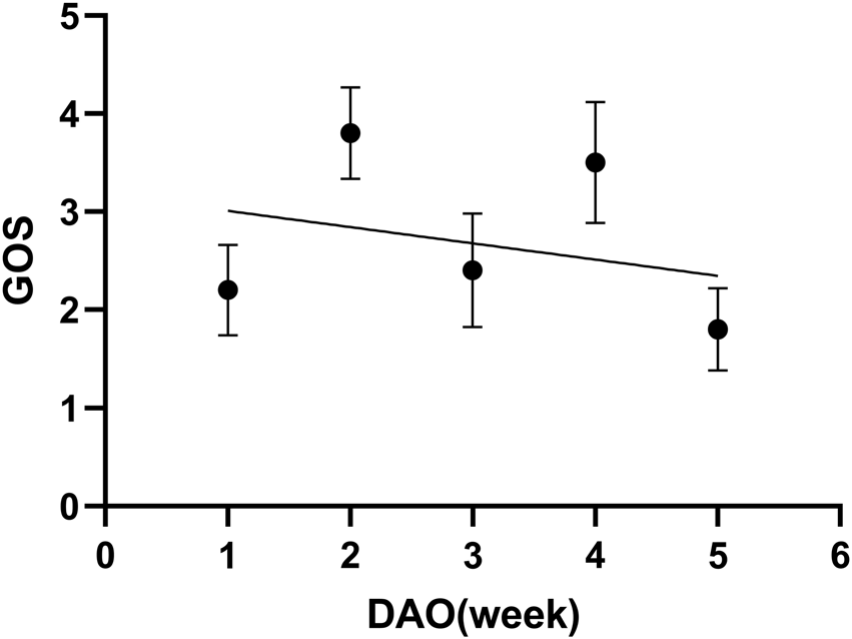
The correlation between the patients’ outcome and the timing of mNGS. DAO: day after onset.

Spearman’s correlation analysis of the relationship between the time of mNGS and GOS suggested the *r* = −0.30, (*P* = 0.68). The time of taking mNGS since onset had an insignificant negative correlation with GOS after 90-day follow-up (Figure 4).

## 4 Discussion

There is intensive interest of mNGS in identifying pathogens of infectious diseases, including CNS infection. Most of previous studies focused on the accuracy of mNGS in identifying pathogens in comparison with conventional microbiological testing [1,5,6,10]. Clinicians usually recognize suspicious CNS infection by symptoms and prescribe antibiotics empirically at the beginning; then, they adjust antibiotics according to routine CSF testing subsequently. Since the empirical antibiotic treatment of CNS infection often cover the majority of common bacteria and viruses, sometimes even *Mycobacterium tuberculosi*s, whether taking mNGS could still benefit the patients with severe CNS infection is the question of our interest. In this study, we investigated the effectiveness of mNGS in terms of diagnosis and antibiotic treatment. We found that mNGS did promote the pathogen identification as well as the accurate antibiotic treatment of critical CNS infection even empirical diagnosis and antibiotics had been established according to clinical manifestations and conventional laboratory testing.

CNS infection has overlapping clinical and laboratory characteristics with other neurological diseases [5]. Furthermore, even the diagnosis of CNS infection is determined. It is difficult to identify the spectrum of pathogen due to the atypical CSF characteristics in many severe cases. The empirical judgment on pathogens may easily lead to mistakes (Figure 2). mNGS in this study has helped to identify the pathogens for 15 cases in which the specific spectrum of pathogens was difficult to be determined by conventional CSF testing and thoroughly changed the empirical judgment on pathogen spectrum for another 12 cases. In addition, it has expanded the pathogen spectrum for 2 cases. These rare pathogens identified by mNGS in severe CNS infections include parasites, *Cryptococcus*, *Coccidioides*, *Rhizopus*, *Aspergillus*, *Mycobacteria*, and so on [11,12]. These pathogens were hard to be detected by routine laboratory tests and some may even out of the consideration of physicians. mNGS takes the advantage of target-independent screening to identify all known pathogens in a sample and elicits great effect on the quick identification of unexpected microbes responsible for severe CNS infection. The effectiveness of mNGS in disclosing the rare intracranial pathogens has been verified by a serial of cases previously reported [13]. The appropriate threshold value of the species-specific read number or genus-specific read numbers for different pathogens has been proposed in order to minimize the false positive findings [1]. It has also acknowledged that mNGS has a relatively high positive prognostic value between 15.7 and 57%, as well as the sensitivity of 73% and the specialty of 99% [1,5,6,13]. Our study provided similar result and supported the value of mNGS in detecting pathogens of severe CNS infection. The overall accuracy of detection of the study was 81.0%, and the sensitivity was even as high as 82.4% while the specificity was 78.6%.

Furthermore, the investigation on the value of mNGS in real practice should go beyond the identification of pathogens. The empirical antibiotic treatment is usually initiated at the moment of CNS infection was suspected and might be adjusted according to the routine CSF analysis followed. Therefore, the practical value of mNGS also concerns whether its merit in accurate identification of pathogens could help to optimize the antibiotic treatment in real practice, because the routine CSF tests, such as the measurement of CSF pressure, the cytology, and biochemistry of CSF, have provided etiological cues a little earlier than mNGS. Given the atypical symptoms of CNS infection and low sensitivity and specificity of conventional CSF testing, great bias in pathogen judgment may exist, resulting in failure in covering pathogens. Even the pathogens were covered, the combined broad-spectrum antibiotics, which used to be empirically applied against suspicious CNS infection, may lead to the abuse of antibiotics [14,15,16,17]. In this study, all patients had received empirical antibiotics prior to mNGS results, some even had undergone adjustment according to conventional CSF tests before mNGS results. However, there were still nearly 50% patients benefited from mNGS in terms of optimizing antibiotic therapy. Furthermore, among the patients with positive mNGS results, promotion of antibiotic treatment by mNGS has been observed in 60.4% cases (Figure 1), mainly toward precision of antibiotics and getting rid of unnecessary drugs. Moreover, the previous study has suggested that the negative mNGS results can help rule out infections, especially in immunocompromised patients [5]. In this study, 29.0% of mNGS-negative cases can be benefited from the de-escalation of empirical antibiotic regimen. Thus, mNGS facilitated the accurate antibiotic treatment by identifying pathogens in severe CNS infection.

In this study, six cases were found with false-positive mNGS detection. The positives were attributed to sample contamination from the environment or normal human flora. Fortunately, the diagnosis and treatment of all these cases were not misled by mNGS owing to the reference of clinical data and conventional laboratory testing (Tables 1 and 3). Eight cases were found false negative; compartmentalized infection (abscess from inner ear) was an important reason for these negative mNGS findings. Antibiotic treatment before mNGS may also contribute to the negative results. In addition, the timing of mNGS and the low abundance or even absence of some specific pathogens in CSF (such as *Borrelia burgdorferi*) may lead to false-negative mNGS results [5,8]. In the study, 74.2% cases with negative mNGS remained their antibiotic treatment with consideration of the complicated infection of other organs or occult infection, especially when the results of mNGS and autoimmune antibodies were both negative [3,5,18,19]. It is suggested that mNGS should be interpreted with clinical context and clinical reasoning.

The decision about the appropriate timing for mNGS was still considered to be made on a case-by-case basis [8]. A health-care economics modeling study based on actual insurance payments for hospitalized patients with CNS infection found that an opportunity exists for mNGS testing to be cost-effective in patients who are critically ill [20].

The follow-up was carried out at the 90th day after discharge of NICU and suggested that the overall mortality of severe CNS infection was as high as 37.1% while the severe disability reached 18.6%. The GOS score of the 90-day follow-up has indicated a negative correlation with the timing of mNGS since onset, but with no statistical significance. Theoretically, the early mNGS is taken, the earlier pathogens are identified, and specific antibiotics could be administrated, avoiding abuse of antibiotics and its complications [21,22]. There are three reasons for the insignificant correlation between the timing of mNGS and the patients’ outcome. On one hand, the rapid progression of severe CNS infection may result in relatively high mortality and disability even receiving early mNGS diagnosis and specific treatment. On the other hand, the mNGS may be delayed due to the consideration on expense in less severe cases which presented better outcome. Therefore, the later mNGS was carried out, the lower GOS score at the 90th day was (Figure 4). Besides, the sample size of this study may be too small to reveal the significance. Previous studies showed that the clinical outcome of severe CNS patients is more affected by systemic status than by the dysfunction of CNS. The early definitive diagnosis and precision medicine may lead to systemic benefit [15,16,22]. Therefore, we still recommend taking mNGS as early as possible in the context of severe CNS infection.

This study suggested that mNGS is of important strength in promoting the accuracy of diagnosis and antibiotic treatment of severe CNS infection, even with the pre-establishment of empirical judgment on pathogens and empirical antibiotic treatment before mNGS. In the severe CNS infection cases, mNGS, as a powerful assistance to clinicians, facilitated the identification of pathogens and improved the accurate antibiotic treatment. It should be taken as early as possible when patients are suspected as severe CNS infection.
